# Supplementary material for: Unique architecture of thermophilic archaeal virus APBV1 and its genome packaging
Source: Nat Commun. 2017 Nov 10;8:1436. doi: 10.1038/s41467-017-01668-0 (PMC5681674; doi:10.1038/s41467-017-01668-0)
Supplement: Supplementary file 1 — Supplementary Information [file 41467_2017_1668_MOESM1_ESM.pdf]

Supplementary Figures

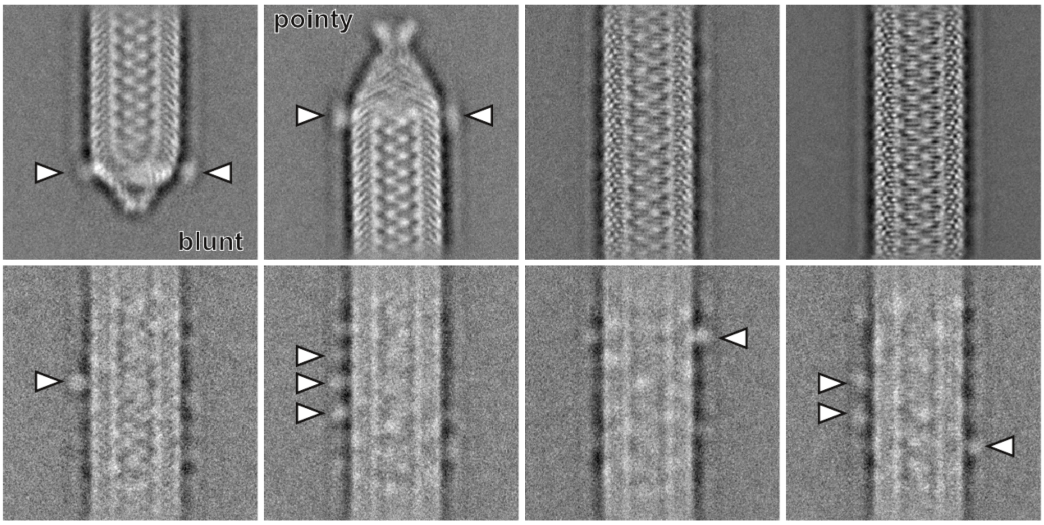

Supplementary Figure 1 | Classification of APBV1 segments extracted from cryo-EM images. Representative two-dimensional class averages of APBV1 segments. Averages showing the blunt and pointy ends of the capsid have been labeled. The arrows indicate protruding densities that were consistently observed at both of the ends and occasionally also in other areas of the capsid.

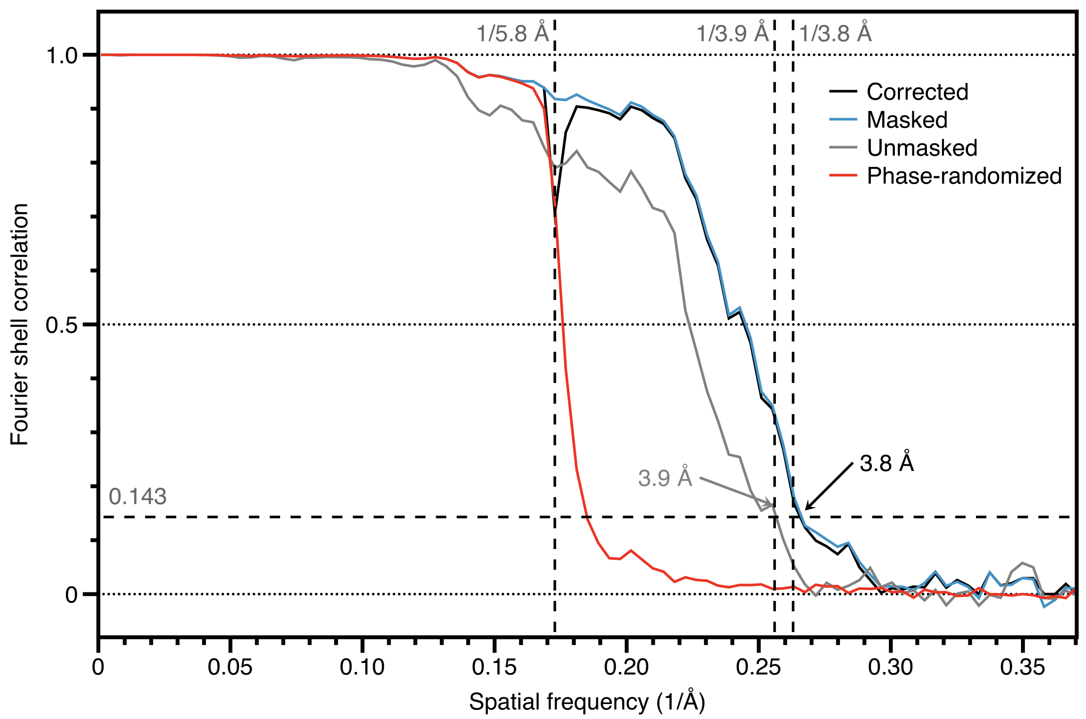

Supplementary Figure 2 | Resolution of the APBV1 helical reconstruction. Fourier shell correlation (FSC) calculated between two half-maps as a function of spatial frequency is plotted. FSC is plotted for the original, unmasked half-maps (grey) and masked half-maps having density corresponding to DNA and solvent removed (blue). FSC is also shown for phase-randomized half-maps (red) where phases were randomized at frequencies higher than 1/5.8 Å. The correlation drops at the cut-off frequency sharply below the noise threshold (0.143) as expected. The phase-randomization test was used to take into account the effect masking on the half-maps before calculating the final corrected FSC curve (black). Good agreement between the masked and corrected curves indicated no adverse effects from masking. The corrected curve drops below the noise threshold at 1/3.8 Å indicating resolution of 3.8 Å in the reconstruction.

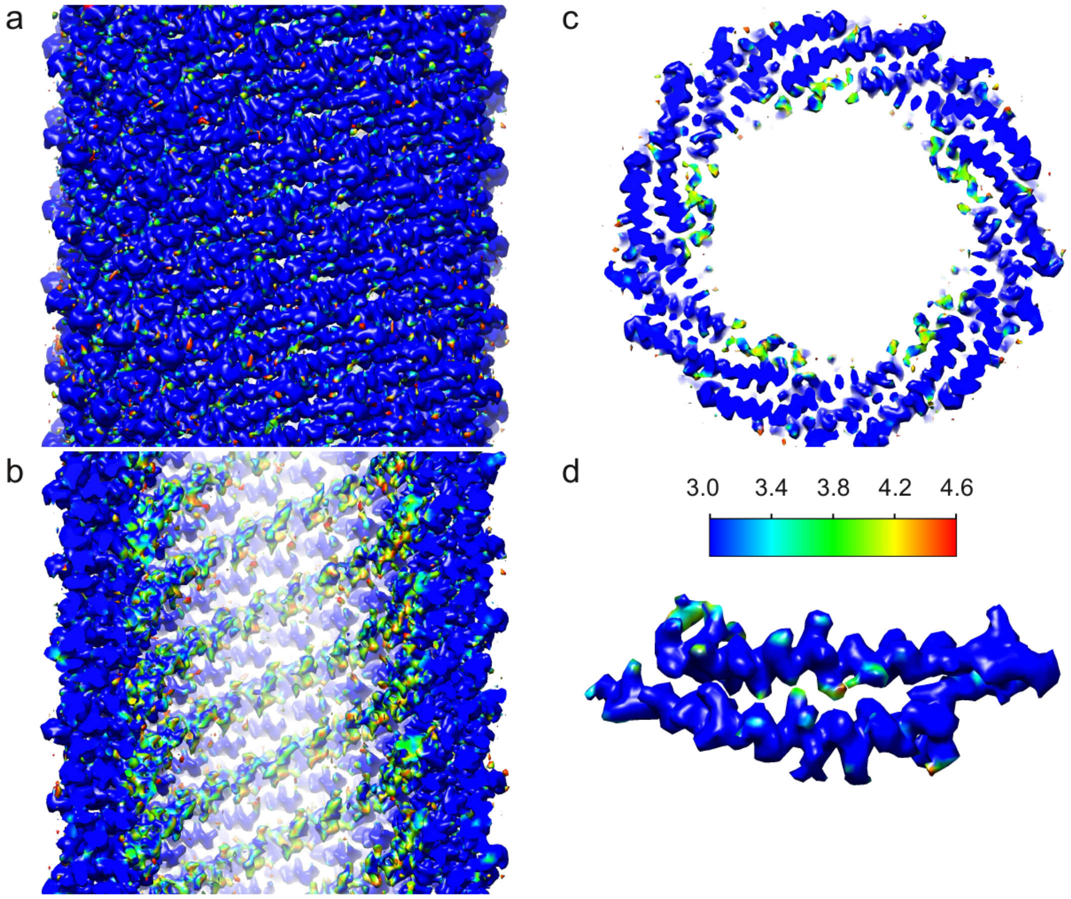

Supplementary Figure 3 | Local resolution analysis. a, APBV1 cryo-EM reconstruction is shown from the side. **b**, The same view as in a is shown with the front half of the reconstruction removed to show internal density. **c**, A slab of density, orthogonal to the long axis of the capsid is shown. **d**, Extracted density corresponding to one VP1 submit is shown. Local resolution in the APBV1 capsid reconstruction (Å) is depicted using a coloured surface as indicated in the coluorbar. The same colouring is used in *a–d*. Most of the VP1 density is resolved to 3.0 Å and the internal densities corresponding to VP1 termini and DNA are resolved at a lower resolution.

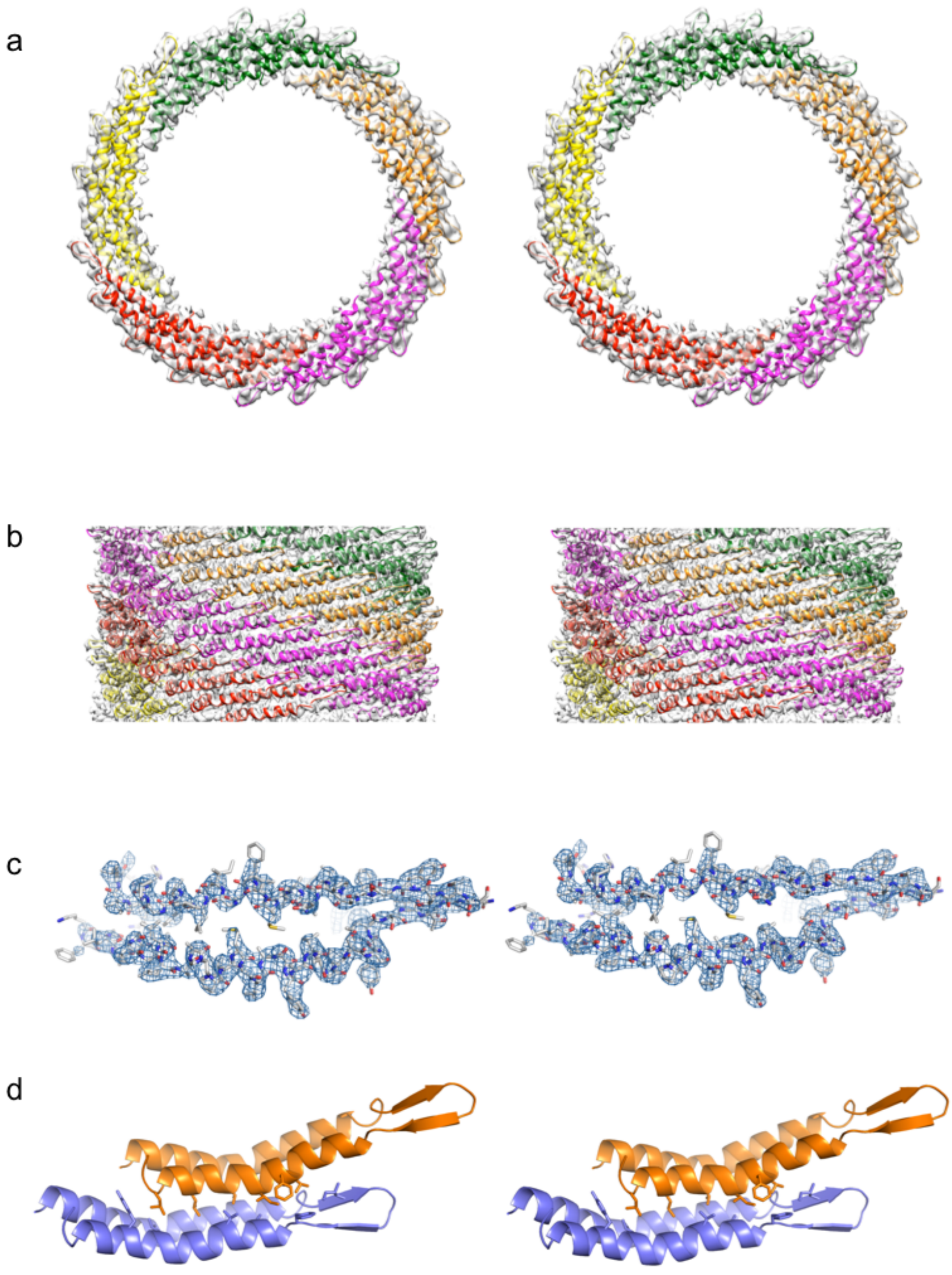

Supplementary Figure 4 | Stereo images of APBV1 assembly. a–b, The helical arrangement of VP1 subunits is shown as ribbons from the top in *(a)* and from the side in *(b)*. **c**, The cryo-EM density (mesh) and VP1 model (sticks) of VP1 are shown for one VP1 subunit. **d**, Two VP1 molecules are rendered as a ribbon in orange and blue. Side-chains involved in subunit–subunit contacts are rendered as sticks.

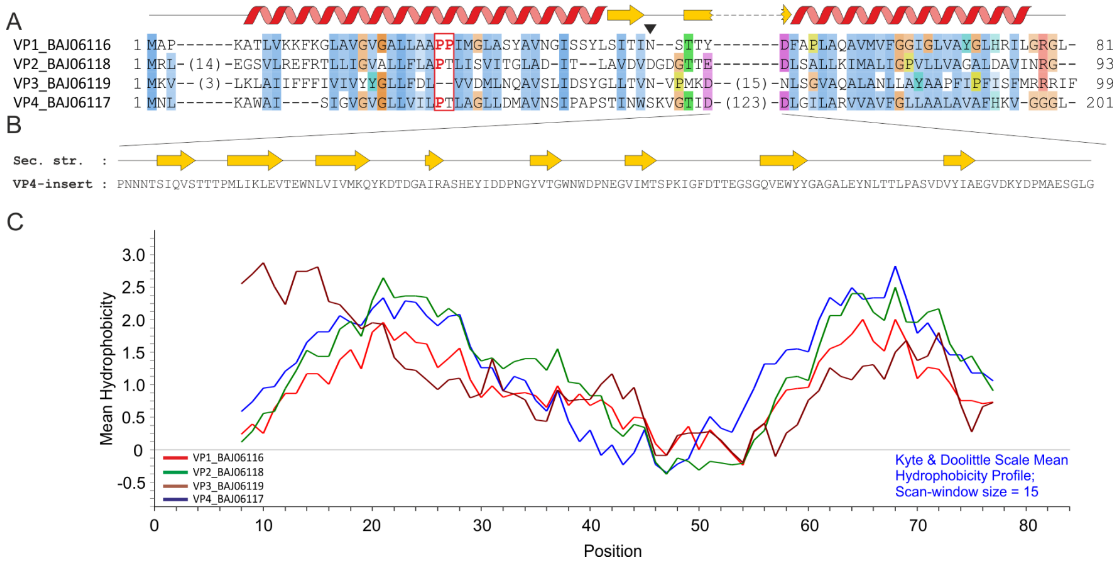

Supplementary Figure 5 | Alignment of APBV1 proteins. a, Protein sequences of the major protein VP1 and minor proteins VP2, VP3 and VP4 are aligned. Secondary structure determined experimentally for VP1 is shown above the corresponding sequence, a potential glycosylation site at Asn47 is indicated with an arrow and proline residues causing a kink in the helix are boxed. **b**, the predicted secondary structure of VP4 fragment containing residues 49–172. **c**, Hydrophobicity plot showing the distribution of hydrophobic and hydrophilic residues in APBV1 proteins. Residue numbering is based on the sequence of VP1. Insertions in VP3 and VP4 have been removed for clarity.

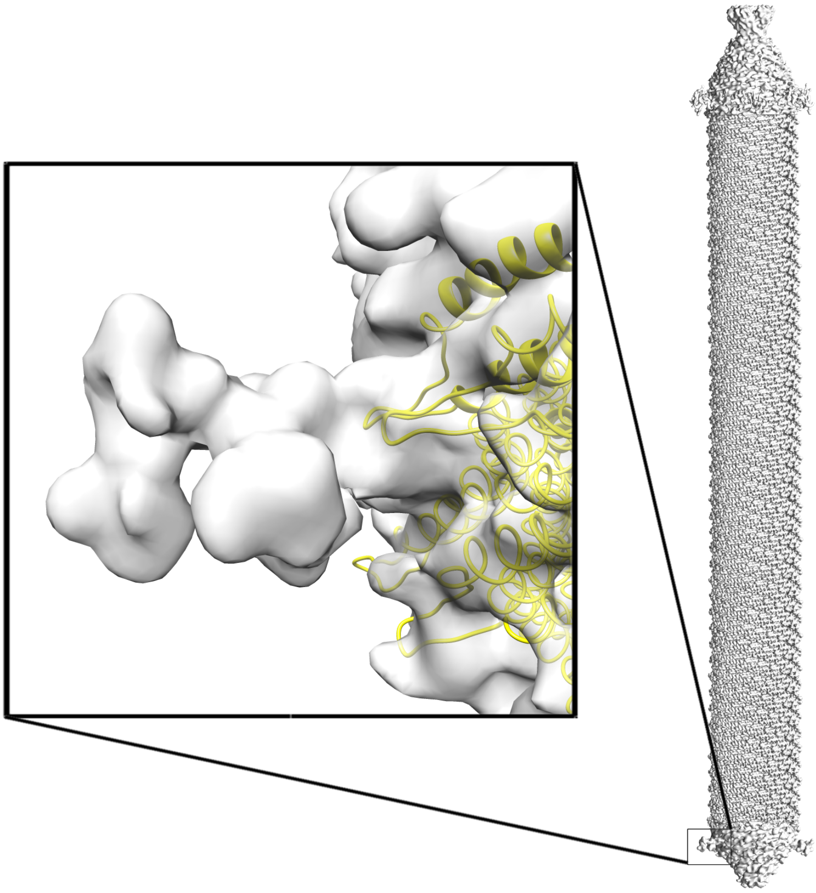

Supplementary Figure 6 | Putative glycosylation in VP1. A close-up of the APBV1 'blunt' tip cryo-EM map is shown to highlight the protruding density corresponding to a putative glycosylation. The VP1 model has been docked in the map (yellow). The Asn47 predicted to be the glycosylation site is located at the origin of the density assigned as glycan. Relatively low resolution of the map (7.1 Å) did not allow accurate modeling of the glycan.

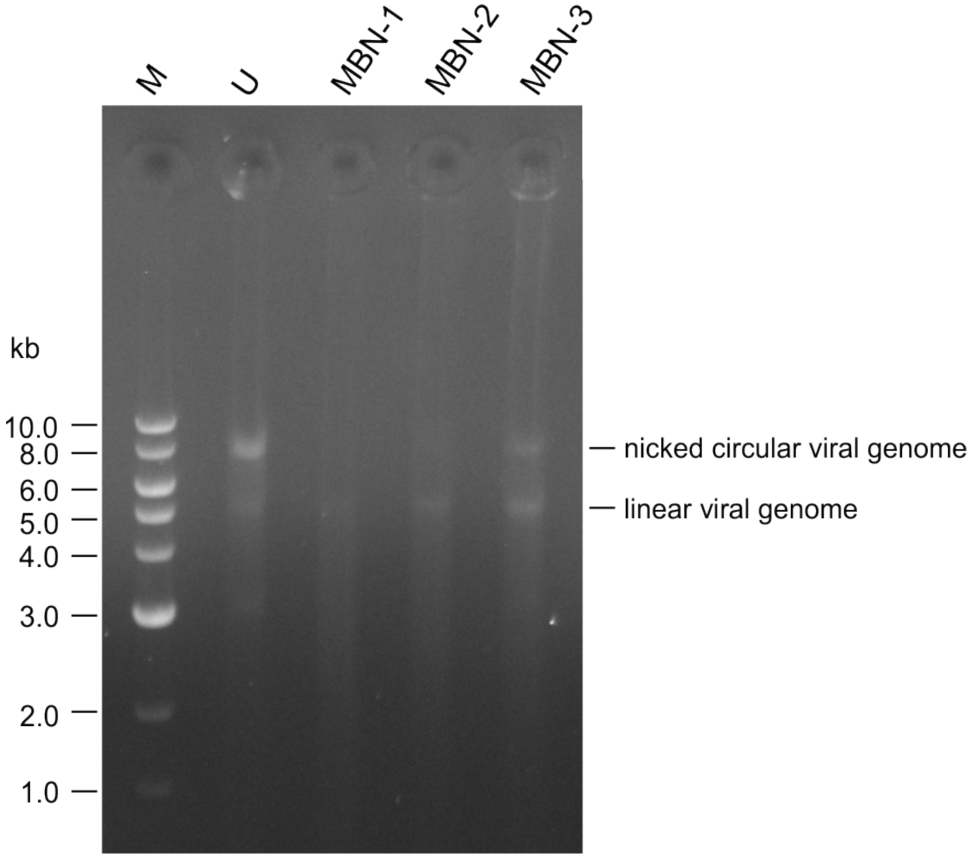

Supplementary Figure 7 | Determination of the superhelical state of the genome. APBV1 genome was treated with mung bean nuclease (MBN) and the products of reactions were separated on 1% agarose gel stained with SYBR Safe (U: undigested genomic DNA control; MBN-1: 30 U/μg DNA, MBN-2: 20 U/μg DNA, and MBN-3: 10 U/μg DNA).

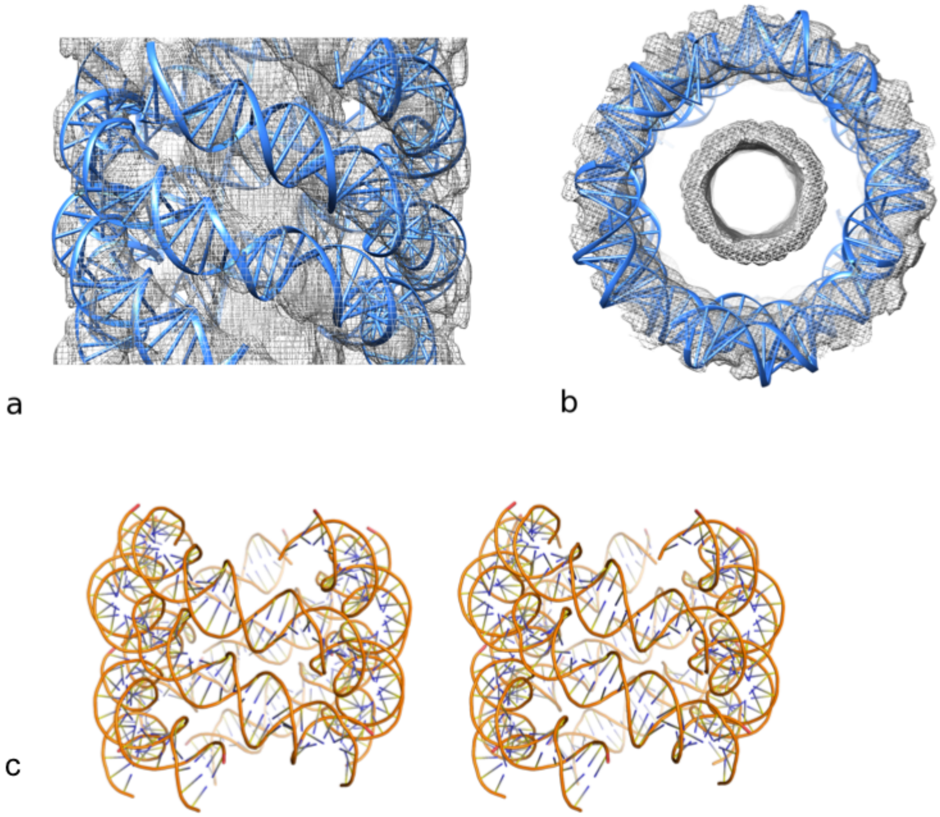

Supplementary Figure 8 | Low pass-filtered map of the APBV1 DNA. a–b, Side (*a*) and top (*b*) view of reconstructed APBV1 DNA low pass-filtered to 8-Å resolution. DNA follows five helical densities proximal to the helical capsid. The sixth pass (not rendered) is predicted to go along the central axis of the virion. **c**, The same view as in *(a)* is shown as a stereo image.

Supplementary Tables

Supplementary Table 1. Data collection statistics.

Parameter	Value
Voltage, (kV)	300
Magnification, ×	37,037
Defocus, μm	1.0–4.2
Dose rate, e^{-}/pixels/s	8
Frames	22
Frame length, s	0.2
Micrographs	400

Supplementary Table 2. Data processing statistics.

Parameter	Filament	Pointy end	Blunt end
Pixel size, Å	1.35	1.35	1.35
Frames used (first–last)	2–5	1–22	1–22
Final dose (total dose) e^{-}/Å^{2}	5 (22)	22 (22)	22 (22)
Particles	3,145	3,145	3,145
Defocus, μm	1.0–2.2	1.0–4.2	1.0–4.2
Segments (total segments)*	94,645 (169,316)	2,572 (6,290)	2,952 (6,290)
Segment overlap, Å	27 Å	N/A	N/A
Box size, pixels	180	320	256
Cyclic symmetry	C5	C5	C5
Helical turn, degrees	–16.55	N/A	N/A
Helical rise, Å	6.10	N/A	N/A
Resolution, Å	3.8	7.2	7.1
B-factor, Å^{2}	116	190	205

* A subset of segments was selected from all segments based on cross-correlation coefficient and 2D classification. Those segments with a low cross-correlation coefficient or those that were assigned into poor 2D classes were excluded.

Supplementary Table 3. VP1 model refinement and validation statistics.

Parameter	Value
**Model composition:**	
Non-hydrogen atoms	504
Protein residues	70
Sugars	0
**Refinement:**	
Resolution (Å)	3.0
Map sharpening factor (Å^2)	–50
Fourier shell correlation (FSC)	0.83
R-factor (%)	27.1
**Rms deviations:**	
Bonds (Å)	0.012
Angles (°)	1.98
***B*-factors**	
Protein (all; Å^2)	86
**Molprobity validation:**	
Clashscore, all atoms	10.65
Molprobity score	2.17
Rotamers favoured (poor) (%)	72.0 (2.0)
Ramachandran plot (% favoured)	94.1
Ramachandran plot (% allowed)	5.9
Ramachandran plot (% outliers)	0.0
